# Supplementary material for: The β-hairpin of 40S exit channel protein Rps5/uS7 promotes efficient and accurate translation initiation in vivo
Source: eLife. 2015 Jul 2;4:e07939. doi: 10.7554/eLife.07939 (PMC4513230; doi:10.7554/eLife.07939)
Supplement: Supplementary file 1. — Phenotypes of RPS5 mutants. DOI: http://dx.doi.org/10.7554/eLife.07939.017 [file elife07939s001.docx]

**Supplementary File 1. Phenotypes of *RPS5* mutants**

|  |  |  | Suppression of His^+^ phenotype^d^ | | Suppression of *SUI5* Slg^-e^ |
| --- | --- | --- | --- | --- | --- |
| *RPS5* allele^a^ | Rel. growth^b^ | Relative UUG:AUG  *HIS4-lacZ*  expression^c^ | *SUI3-2* | *SUI5* |  |
| WT | 4 | 1 | N | N | N |
| *E144A* | 3 | 1.3±0.003 | N |  |  |
| *E144R* | 1 | 5.9±0.02 | N |  |  |
| *E144A D145A* | 3 | 2.0±0.006 | N |  |  |
| *D145A* | 4 | 0.9±0.003 | N |  |  |
| *T146A T147A* | 4 | 0.7±0.001 | N |  |  |
| *R148A* | 4 | 0.8±0.002 | Y | Y | Y |
| *R148E* | 4 |  | Y | Y | Y |
| *G151R* | 4 | 0.8±0.002 | N |  |  |
| *G151S* | Lethal | - | - | - | - |
| *G152A* | 4 | 1.0±0.002 | N |  |  |
| *G152S* | 4 | 0.9±0.002 | N |  |  |
| *G152D* | 3 | 1.2±0.002 | N |  |  |
| *G152K* | 4 | 0.6±0.001 | N |  |  |
| *G153A* | 4 | 0.4±0.001 | Y | Y | Y |
| *G153K* | 3 | 1.4±0.002 | N |  |  |
| *A154T* | 4 | 0.9±0.003 | N |  |  |
| *A154R* | 4 | 0.3±0.001 | Y | Y | Y |
| *A155E* | 4 | 0.5±0.001 | Y | Y | Y |
| *A155V* | 4 | 0.7±0.004 | N |  |  |
| *A155R* | 4 | 0.7±0.001 | N |  |  |
| *R156A* | 4 | 0.7±0.002 | Y | Y | Y |
| *R156E* | 4 |  | Y | Y | Y |
| *R157A* | 4 | 0.9±0.004 | Y | Y | Y |
| *R157E* | 4 |  | Y | Y | Y |
| *Q158A* | 4 | 1.2±0.005 | N |  |  |
| *K222A* | 4 | 0.6±0.002 | Y | Y | Y |
| *S223A* | 2 | 2.0±0.008 | N |  |  |
| *N224A* | 4 | 1.2±0.008 | N |  |  |
| *R225K* | 2 | 3.6±0.005 | N |  |  |
| *R225E* | 2 | 2.9±0.005 | N |  |  |
| *R225A* | 3 | 2.8±0.006 | N |  |  |
| *E144R R225E* | 2 | 4.3±0.01 | N |  |  |

^a^Indicated *RPS5* alleles on lc *LEU2* vector pRS315.

^b^Transformants of strain JVY07 (*P_GAL1-_RPS5*) containing the indicated plasmid-borne *RPS5* alleles were streaked on SD+His+Ura+Trp and incubated at 30°C for 2da. Relative colony growth was judged qualitatively, with growth similar to the WT *RPS5* strain scored as 4 and very slow-growth scored as 1.

^c^Ratio of expression of matched *HIS4-lacZ* reporters differing only at their start codons, UUG or AUG, measured as described in Figure 3B, and expressed relative to WT *RPS5* with the indicated S.E.M.s.

^d^Ability of the *RPS5* allele to suppress the His^+^ phenotype of *his4-301(ACG)* conferred by *SUI3-2* or *SUI5*, indicated as yes (Y) or no (N).

^e^Ability of the *RPS5* allele to suppress the Slg^-^ phenotype conferred by *SUI5*, indicated as yes (Y) or no (N).
