# Supplementary material for: The β-hairpin of 40S exit channel protein Rps5/uS7 promotes efficient and accurate translation initiation in vivo
Source: eLife. 2015 Jul 2;4:e07939. doi: 10.7554/eLife.07939 (PMC4513230; doi:10.7554/eLife.07939)
Supplement: Supplementary file 2. — Yeast strains employed in this study. DOI: http://dx.doi.org/10.7554/eLife.07939.018 [file elife07939s002.docx]

**Supplementary File. 2 Yeast strains employed in this study.**

| Strain | Genotype | Source or reference |
| --- | --- | --- |
| HLV01a | *MATa ura3-52 trp1Δ-63 leu2-3,112 his4-301(ACG)* | Leos Valasek |
| H4564 | *MATa ura3-52 trp1Δ-63 leu2-3,112 his4-301(ACG) sui1Δ::hisG* pPMB03 (sc *LEU2 sui1-L96P*) | {Martin-Marcos, 2011 #6875} |
| JVY07 | *MATa ura3-52 trp1Δ-63 leu2-3,112 his4-301(ACG) kanMX6:P_GAL1_-RPS5* | This study |
| JVY31 | *MATa ura3-52 trp1Δ-63 leu2-3,112 his4-301(ACG) kanMX6:P_GAL1_-RPS5* pJV09 (lc *LEU2 RPS5*) | This study |
| JVY32 | *MATa ura3-52 trp1Δ-63 leu2-3,112 his4-301(ACG) kanMX6:P_GAL1_-RPS5* pJV09 (lc *LEU2 rps5-E144R*) | This study |
| JVY33 | *MATa ura3-52 trp1Δ-63 leu2-3,112 his4-301(ACG) kanMX6:P_GAL1_-RPS5* pJV09 (lc *LEU2 rps5-R225K*) | This study |
| JVY56 | *MATa ura3-52 trp1Δ-63 leu2-3,112 his4-301(ACG) kanMX6:P_GAL1_-RPS5* pJV09 (lc *LEU2 rps5-R148A*) | This study |
| JVY57 | *MATa ura3-52 trp1Δ-63 leu2-3,112 his4-301(ACG) kanMX6:P_GAL1_-RPS5* pJV09 (lc *LEU2 rps5-R156A*) | This study |
| JVY58 | *MATa ura3-52 trp1Δ-63 leu2-3,112 his4-301(ACG) kanMX6:P_GAL1_-RPS5* pJV09 (lc *LEU2 rps5-R157A*) | This study |
| JVY59 | *MATa ura3-52 trp1Δ-63 leu2-3,112 his4-301(ACG) kanMX6:P_GAL1_-RPS5* pJV09 (lc *LEU2 rps5-A154R*) | This study |
| JVY60 | *MATa ura3-52 trp1Δ-63 leu2-3,112 his4-301(ACG) kanMX6:P_GAL1_-RPS5* pJV09 (lc *LEU2 rps5-A155E*) | This study |
| JVY61 | *MATa ura3-52 trp1Δ-63 leu2-3,112 his4-301(ACG) kanMX6:P_GAL1_-RPS5* pJV09 (lc *LEU2 rps5-R148E*) | This study |
| JVY62 | *MATa ura3-52 trp1Δ-63 leu2-3,112 his4-301(ACG) kanMX6:P_GAL1_-RPS5* pJV09 (lc *LEU2 rps5-R156E*) | This study |
| JVY63 | *MATa ura3-52 trp1Δ-63 leu2-3,112 his4-301(ACG) kanMX6:P_GAL1_-RPS5* pJV09 (lc *LEU2 rps5-R157E*) | This study |
| F2009/YSC1021-672858 | *MATa/MATα ura3-∆0/ura3-∆0 leu2-∆0/leu2-∆0 his3∆-1/his3∆-1 lys2-∆0/LYS2 met15-∆0/MET15 rps5∆::kanMX/RPS5* | Open Biosystems |
| JVY11 | *MATα ura3-∆0 leu2-∆0 his3∆-1 lys2-∆0 MET15 rps5∆::kanMX* pJV38 (lc *URA3 RPS5)* | This study |
| JVY15 | *MATα ura3-∆0 leu2-∆0 his3∆-1 lys2-∆0 MET15 rps5∆::kanMX* pJV13 (lc *LEU2 rps5-E144R)* | This study |
| JVY29 | *MATα ura3-∆0 leu2-∆0 his3∆-1 lys2-∆0 MET15 rps5∆::kanMX* pJV09 (lc *LEU2 RPS5)* | This study |
| JVY52 | *MATα ura3-∆0 leu2-∆0 his3∆-1 lys2-∆0 MET15 rps5∆::kanMX* pJV35 (lc *LEU2 rps5-R148E)* | This study |
