# Supplementary material for: The β-hairpin of 40S exit channel protein Rps5/uS7 promotes efficient and accurate translation initiation in vivo
Source: eLife. 2015 Jul 2;4:e07939. doi: 10.7554/eLife.07939 (PMC4513230; doi:10.7554/eLife.07939)
Supplement: Supplementary file 3. — Plasmids employed in this study. DOI: http://dx.doi.org/10.7554/eLife.07939.019 [file elife07939s003.docx]

**Supplementary File 3. Plasmids employed in this study.**

| Plasmid | Description^a^ | Source or reference |
| --- | --- | --- |
| pRS315 | lc *LEU2* cloning vector | {Sikorski, 1989 #1817} |
| pRS316 | lc *URA3* cloning vector | {Sikorski, 1989 #1817} |
| pFA6a-kanMX6-P_GAL1_ | Inducible *GAL1* promoter insertion cassette | {Longtine, 1998 #4292} |
| pJV01 | lc *LEU2 RPS5* in pRS315 | This study |
| pJV09 | lc *LEU2 RPS5* with BglII site engineered in pJV01 | This study |
| pJV38 | lc *URA3* *RPS5* with BglII site engineered in pRS316 | This study |
| pJV12 | lc *LEU2* *rps5-E144A* | This study |
| pJV13 | lc *LEU2* *rps5-E144R* | This study |
| pJV14 | lc *LEU2* *rps5-D145A* | This study |
| pJV15 | lc *LEU2* *rps5-R148A* | This study |
| pJV16 | lc *LEU2* *rps5-G151S* | This study |
| pJV17 | lc *LEU2* *rps5-G151R* | This study |
| pJV18 | lc *LEU2* *rps5-G152A* | This study |
| pJV19 | lc *LEU2* *rps5-G152S* | This study |
| pJV20 | lc *LEU2* *rps5-G152D* | This study |
| pJV21 | lc *LEU2* *rps5-G152K* | This study |
| pJV22 | lc *LEU2* *rps5-G153A* | This study |
| pJV23 | lc *LEU2* *rps5-G153K* | This study |
| pJV24 | lc *LEU2* *rps5-A154T* | This study |
| pJV25 | lc *LEU2* *rps5-A154R* | This study |
| pJV26 | lc *LEU2* *rps5-A155V* | This study |
| pJV27 | lc *LEU2* *rps5-A155R* | This study |
| pJV28 | lc *LEU2* *rps5-A155E* | This study |
| pJV29 | lc *LEU2* *rps5-R156A* | This study |
| pJV30 | lc *LEU2* *rps5-R157A* | This study |
| pJV31 | lc *LEU2* *rps5-Q158A* | This study |
| pJV32 | lc *LEU2* *rps5-K222A* | This study |
| pJV33 | lc *LEU2* *rps5-S223A* | This study |
| pJV34 | lc *LEU2* *rps5-N224A* | This study |
| pJV35 | lc *LEU2* *rps5-R225K* | This study |
| pJV36 | lc *LEU2* *rps5-ED144-145AA* | This study |
| pJV37 | lc *LEU2* *rps5-TT146-147AA* | This study |
| pJV39 | lc *LEU2* *rps5-R148E* | This study |
| pJV40 | lc *LEU2* *rps5-R156E* | This study |
| pJV41 | lc *LEU2* *rps5-R157E* | This study |
| pJV51 | lc *LEU2* *rps5-R225E* | This study |
| pJV52 | lc *LEU2* *rps5-R225A* | This study |
| pJV53 | lc *LEU2* *rps5-E144R R225K* | This study |
| p367 | sc *URA3 HIS4(ATG)-lacZ* | {Donahue, 1988 #1344} |
| p391 | sc *URA3 HIS4(TTG)-lacZ* | {Donahue, 1988 #1344} |
| p180 | sc *URA3 GCN4-lacZ* in YCp50 | {Hinnebusch, 1985 #1277} |
| p4280/YCpSUI3-S264Y-W | sc *TRP1 SUI3-S264Y* in YCplac22 | {Valasek, 2004 #5005} |
| p4281/YCpTIF5-G31R-W | sc *TRP1 TIF5-G31R* in YCplac22 | {Valasek, 2004 #5005} |
| pPMB21 | sc *TRP1 SUI1* in YCplac22 | {Martin-Marcos, 2011 #6875} |
| pPMB24 | sc *URA3 SUI1-lacZ* | {Martin-Marcos, 2011 #6875} |
| pPMB25 | sc *URA3 SUI1-opt-lacZ* | {Martin-Marcos, 2011 #6875} |
| pC3502 | sc *URA3* ^-3^AAA^-1^ el.uORF1 *GCN4-lacZ* in YCp50^b^ | Pittman and Dever (unpublished) |
| pC4466 | sc *URA3* ^-3^UAA^-1^ el.uORF1 *GCN4-lacZ* in YCp50^b^ | Pittman and Dever (unpublished) |
| pC3503 | sc *URA3* ^-3^UUU^-1^ el.uORF1 *GCN4-lacZ* in YCp50^b^ | Pittman and Dever (unpublished) |
| pC3505 | sc *URA3* el.uORF1-less *GCN4-lacZ* in YCp50^b^ | Pittman and Dever (unpublished) |

^a^lc, low copy number; sc, single copy.

^b^Derived from pM226 {Grant, 1994 #2381} in which a segment of the *GCN4* mRNA leader extending from the 3’ end of uORF1 to the 3’end of uORF4 is missing, moving uORF1 into the normal position of uORF4, and a frameshift mutation is present in the uORF1 stop codon together with mutations in two stop codons in the uORF4-*GCN4* interval and beginning of the *GCN4* coding sequence, which elongate uORF1 to a 93-codon uORF that overlaps the beginning of *GCN4*  by ~130 nt. The sequences of the three nucleotides immediately upstream of the uORF1 AUG codon were modified in pM226, as indicated for pC3502, pC4466 and pC3503, or the uORF1 AUG was modified to AGC for pC3505, and *lacZ* was fused to the *GCN4* coding sequences.
