# Supplementary material for: The β-hairpin of 40S exit channel protein Rps5/uS7 promotes efficient and accurate translation initiation in vivo
Source: eLife. 2015 Jul 2;4:e07939. doi: 10.7554/eLife.07939 (PMC4513230; doi:10.7554/eLife.07939)
Supplement: Supplementary file 4. — Oligonucleotide primers employed for mutagenesis in this study. DOI: http://dx.doi.org/10.7554/eLife.07939.020 [file elife07939s004.docx]

**Supplementary File 4. Oligonucleotide primers employed for mutagenesis in this study^1^.**

| Name | Sequence | Mutation |
| --- | --- | --- |
| E144A | AACACTGGTCCAAGAGCAGACACCACCAGAGTC | E144A |
| E144A_r | GACTCTGGTGGTGTCTGCTCTTGGACCAGTGTT | E144A |
| E144R | CGCTATCACCAACACTGGTCCAAGAAGAGACACCACCAGA | E144R |
| E144R_r | TCTGGTGGTGTCTCTTCTTGGACCAGTGTTGGTGATAGCG | E144R |
| D145A | CTGGTCCAAGAGAAGCCACCACCAGAGTCGG | D145A |
| D145A_r | CCGACTCTGGTGGTGGCTTCTCTTGGACCAG | D145A |
| R148A | AGAGAAGACACCACCGCAGTCGGTGGTGGTGG | R148A |
| R148A_r | CCACCACCACCGACTGCGGTGGTGTCTTCTCT | R148A |
| G151S | CACCAGAGTCGGTAGTGGTGGTGCTGC | G151S |
| G151S_r | GCAGCACCACCACTACCGACTCTGGTG | G151S |
| G151R | CACCAGAGTCGGTCGTGGTGGTGCTGC | G151R |
| G151R_r | GCAGCACCACCACGACCGACTCTGGTG | G151R |
| G152A | CAGAGTCGGTGGTGCTGGTGCTGCTAGAC | G152A |
| G152A_r | GTCTAGCAGCACCAGCACCACCGACTCTG | G152A |
| G152S | CCAGAGTCGGTGGTAGTGGTGCTGCTAGA | G152S |
| G152S_r | TCTAGCAGCACCACTACCACCGACTCTGG | G152S |
| G152D | CAGAGTCGGTGGTGATGGTGCTGCTAGAC | G152D |
| G152D_r | GTCTAGCAGCACCATCACCACCGACTCTG | G152D |
| G152K | ACCACCAGAGTCGGTGGTAAGGGTGCTGCTAGACGTC | G152K |
| G152K_r | GACGTCTAGCAGCACCCTTACCACCGACTCTGGTGGT | G152K |
| G153A | GTCGGTGGTGGTGCTGCTGCTAGACGT | G153A |
| G153A_r | ACGTCTAGCAGCAGCACCACCACCGAC | G153A |
| G153K | CACCAGAGTCGGTGGTGGTAAGGCTGCTAGACGTCAAGC | G153K |
| G153K_r | GCTTGACGTCTAGCAGCCTTACCACCACCGACTCTGGTG | G153K |
| A154T | GTCGGTGGTGGTGGTACTGCTAGACGTCAAG | A154T |
| A154T_r | CTTGACGTCTAGCAGTACCACCACCACCGAC | A154T |
| A154R | GAGTCGGTGGTGGTGGTCGTGCTAGACGTCAA | A154R |
| A154R_r | TTGACGTCTAGCACGACCACCACCACCGACTC | A154R |
| A155V | GTGGTGGTGGTGCTGTTAGACGTCAAGCTGT | A155V |
| A155V_r | ACAGCTTGACGTCTAACAGCACCACCACCAC | A155V |
| A155R | GGTGGTGGTGGTGCTCGTAGACGTCAAGCTGT | A155R |
| A155R_r | ACAGCTTGACGTCTACGAGCACCACCACCACC | A155R |
| A155E | GGTGGTGGTGGTGCTGAGAGACGTCAAGCTGTCG | A155E |
| A155E_r | CGACAGCTTGACGTCTCTCAGCACCACCACCACC | A155E |
| R156A | GGTGGTGGTGGTGCTGCTGCACGTCAAGCTGTC | R156A |
| R156A_r | GACAGCTTGACGTGCAGCAGCACCACCACCACC | R156A |
| R157A | TGGTGGTGGTGCTGCTAGAGCTCAAGCTGTCG | R157A |
| R157A_r | CGACAGCTTGAGCTCTAGCAGCACCACCACCA | R157A |
| Q158A | TGGTGGTGCTGCTAGACGTGCAGCTGTCGATGTTTC | Q158A |
| Q158A_r | GAAACATCGACAGCTGCACGTCTAGCAGCACCACCA | Q158A |
| K222A | GATGAATTGGAACGTGTTGCCGCGTCTAACCGTTAAGAAGCTAA | K222A |
| K222A_r | TTAGCTTCTTAACGGTTAGACGCGGCAACACGTTCCAATTCATC | K222A |
| S223A | TGGAACGTGTTGCCAAGGCTAACCGTTAAGAAGCT | S223A |
| S223A_r | AGCTTCTTAACGGTTAGCCTTGGCAACACGTTCCA | S223A |
| N224A | GATGAATTGGAACGTGTTGCCAAGTCTGCCCGTTAAGAAGCTAAAAAAAG | N224A |
| N224A_r | CTTTTTTTAGCTTCTTAACGGGCAGACTTGGCAACACGTTCCAATTCATC | N224A |
| R225A | GGAACGTGTTGCCAAGTCTAACGCTTAAGAAGCTAAAAAAAGTGAA | R225A |
| R225A_r | TTCACTTTTTTTAGCTTCTTAAGCGTTAGACTTGGCAACACGTTCC | R225A |
| R225K | ATGAATTGGAACGTGTTGCCAAGTCTAACAAGTAAGAAGCTAAAAAAAGTGAAAGATTTTC | R225K |
| R225K_r | GAAAATCTTTCACTTTTTTTAGCTTCTTACTTGTTAGACTTGGCAACACGTTCCAATTCAT | R225K |
| R225E | GAATTGGAACGTGTTGCCAAGTCTAACGAGTAAGAAGCTAAAAAAAGTGAAAGATTT | R225E |
| R225E_r | AAATCTTTCACTTTTTTTAGCTTCTTACTCGTTAGACTTGGCAACACGTTCCAATTC | R225E |
| ED144-145AA | CCAACACTGGTCCAAGAGCAGCCACCACCAGAGTCGG | ED144-145AA |
| ED144-145AA_r | CCGACTCTGGTGGTGGCTGCTCTTGGACCAGTGTTGG | ED144-145AA |
| TT146-147AA | GGTCCAAGAGAAGACGCCGCCAGAGTCGGTGGTGG | TT146-147AA |
| TT146-147AA_r | CCACCACCGACTCTGGCGGCGTCTTCTCTTGGACC | TT146-147AA |
| R148E | CAAGAGAAGACACCACCGAGGTCGGTGGTGGTGGTGC | R148E |
| R148E_r | GCACCACCACCACCGACCTCGGTGGTGTCTTCTCTTG | R148E |
| R156E | GGTGGTGGTGGTGCTGCTGAGCGTCAAGCTGTCGATG | R156E |
| R156E_r | CATCGACAGCTTGACGCTCAGCAGCACCACCACCACC | R156E |
| R157E | TGGTGGTGGTGCTGCTAGAGAGCAAGCTGTCGATGTTTCTC | R157E |
| R157E_r | GAGAAACATCGACAGCTTGCTCTCTAGCAGCACCACCACCA | R157E |

^1^Primer sequences are listed in 5’ to 3’ direction
